# The ketogenic diet and metabolic treatments for neuropsychiatric disorders

**DOI:** 10.1192/bjo.2025.50

**Published:** 2025-05-09

**Authors:** Christopher M. Palmer

**Affiliations:** Metabolic and Mental Health Program, McLean Hospital, Belmont, MA, USA; Psychiatry, Harvard Medical School, Boston, MA, USA

**Keywords:** Ketogenic, diet, bipolar, neuropsychiatric, metabolic

## Abstract

The ketogenic diet, initially developed for epilepsy treatment, has gained attention as a potential intervention for neuropsychiatric disorders. A groundbreaking study by Campbell et al highlights its feasibility and potential efficacy in bipolar disorder, shedding light on shared mechanisms across neuropsychiatric disorders and the promise of metabolic treatment approaches.


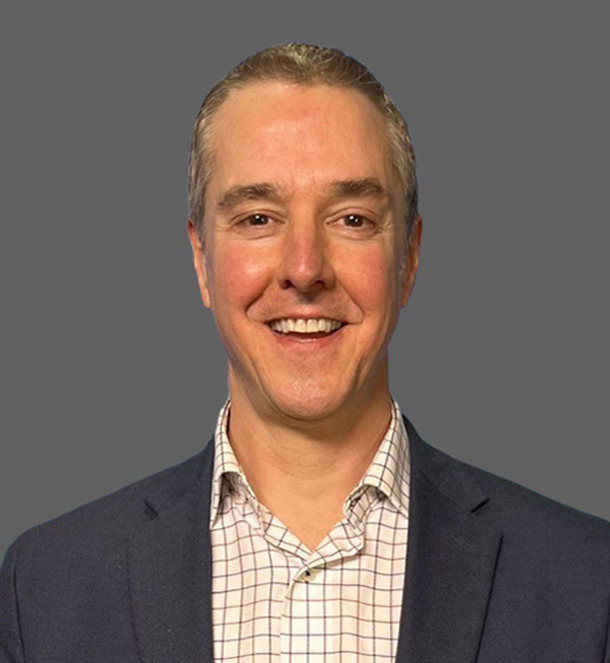


The ketogenic diet, originally introduced over a century ago to treat epilepsy, is low in carbohydrates, moderate in protein and high in fat, and is more commonly recognised today for its roles in weight management and type 2 diabetes. However, emerging evidence suggests that its therapeutic potential extends to a broad range of neuropsychiatric disorders. A pivotal study by Campbell et al, published in *BJPsych Open*, underscores the feasibility and potential efficacy of the ketogenic diet in bipolar disorder.^1^

This research not only validates the ketogenic diet as a viable intervention, but also deepens our understanding of its underlying mechanisms.

## The ketogenic diet: an evidence-based treatment for epilepsy

The therapeutic basis of the ketogenic diet lies in its well-established efficacy for drug-resistant epilepsy (DRE). Since its inception in 1921 by Russell Wilder, the ketogenic diet has demonstrated success in controlling seizures.^2^ A 2020 Cochrane Review synthesised 13 studies involving over 1900 participants, and found that the ketogenic diet was approximately three times more likely to achieve seizure freedom and six times more likely to reduce seizure frequency in children with DRE, compared with standard treatments.^3^ While efficacy in adults is less robust, the evidence remains promising. Over 250 ketogenic diet centres around the world currently offer this therapeutic intervention to thousands of people with DRE.

Research highlights numerous mechanisms through which the ketogenic diet treats epilepsy:^4^

- direct anti-seizure effects of ketone bodies
- neurotransmitter modulation (GABA, glutamate and adenosine)
- ion channel regulation
- enhanced mitochondrial function and bioenergetics
- glycolytic restriction
- reduction of oxidative stress
- direct fatty acid effects
- tricarboxylic acid (TCA) cycle enhancement.

These mechanisms offer insight into how the ketogenic diet might also be used to treat psychiatric conditions.

## Shared pathophysiologies in neuropsychiatric disorders

Mental illnesses such as bipolar disorder, schizophrenia, autism, Alzheimer’s disease and depression share common pathophysiologies. These include mitochondrial dysfunction, glucose hypometabolism, dysregulated insulin signalling, chronic inflammation and oxidative stress.^5^ These abnormalities impair adenosine triphosphate (ATP) production, neurotransmitter and hormone regulation and neuroinflammatory processes, contributing to the pathophysiology and progression of mental illnesses.

The ‘p-factor’, proposed by Caspi et al, posits that psychiatric disorders share common pathophysiological roots. Although there is much debate about the p-factor, whether it exists and what it might be, I and others have proposed that the p-factor may, in fact, be metabolic dysregulation or dysfunction.^6^ Metabolism is influenced by many biopsychosocial factors and allows us to connect most, if not all, of the risk factors for mental illness.

The ketogenic diet addresses many of these shared metabolic abnormalities, positioning itself as a transdiagnostic treatment rather than one limited to individual diagnoses. In fact, over 50 case reports and pilot trials, representing over 1900 participants, have already been published, suggesting that it might be an effective treatment for a wide range of psychiatric disorders.^5^

## Pilot trial and neuroimaging insights

Campbell et al took this work to a new level of rigour in their pilot trial of 20 participants with bipolar disorder. Participants demonstrated high levels of adherence to the ketogenic diet, with 91% of their daily ketone readings being positive.^7^ Given that participants were recruited while in a euthymic state and that this was a six- to eight-week trial, it is unsurprising that there were no significant changes in symptoms as measured by Affective Lability Scale-18, Beck’s Depression Inventory and Young Mania Rating Scale. However, when looking at more subtle and persistent symptoms often experienced by people with bipolar disorder and measured by the daily ecological momentary assessment, there was an association between daily ketone levels and improvements in mood, energy, anxiety and impulsivity. Participants’ metabolic health also improved, with significant weight loss and reductions in systolic blood pressure. Advanced neuroimaging revealed reductions in brain Glx (the combination of glutamate and glutamine) in both the anterior cingulate cortex and posterior cingulate cortex. This measure is associated with reduced excitotoxicity, a feature implicated in both bipolar disorder and epilepsy – consistent with neurotransmitter modulation as one of the mechanisms of action of the ketogenic diet.

## Beyond diet: broader metabolic interventions

At first glance, it is surprising to many that an intervention for epilepsy is also an evidence-based treatment for weight loss and type 2 diabetes.^8,9^ However, metabolic disorders such as obesity, type 2 diabetes and non-alcoholic fatty liver disease also share metabolic dysregulation as a common pathophysiology with neuropsychiatric disorders. These are also common, although certainly not universal, comorbidities in people with mental disorders. The ketogenic diet has demonstrated efficacy in treating all of these conditions through mechanisms such as glucose metabolism optimisation, lipid regulation and gut microbiota changes.^10^ Together, this evidence underscores the potential of the ketogenic diet to address both systemic and neurological dysfunctions simultaneously.

In addition to the ketogenic diet, other metabolic interventions, including pharmacological agents, supplements and lifestyle modifications, hold promise.

For example, there is growing interest in glucagon-like peptide-1 (GLP-1) receptor agonists in the treatment of neuropsychiatric disorders. This class of medication was initially developed to treat type 2 diabetes and is now widely used for weight loss.^11^ Like the ketogenic diet, this treatment holds promise as a treatment for weight loss, diabetes and neuropsychiatric disorders.

Supplements that improve mitochondrial function, such as carnitine, creatine, Co-Q and vitamin B2, also hold promise as adjunctive treatments for neuropsychiatric disorders.^12^

Metabolic approaches such as these could amplify the therapeutic effects of the ketogenic diet while broadening treatment options.

## Challenges and future directions

Despite its promise, several barriers must be addressed for the ketogenic diet to reach its full potential in psychiatric care.

(a) Methodological challenges of dietary research. Dietary research poses many challenges, including frequently small sample size, lack of blinding, variations in baseline exposures and dietary patterns, and appropriate control groups.^13^

(b) Compliance. One of the biggest concerns of clinicians and researchers is compliance. Although adherence to any diet can be difficult, and the ketogenic diet is more restrictive than most, Campbell et al demonstrated that they could achieve high levels of adherence. While they recruited participants in a euthymic state, real-world compliance may vary during mood episodes due to cognitive or motivational challenges.

(c) Safety and side-effects. The long-term safety of the ketogenic diet is debated, with the most commonly cited concerns being increases in total, low-density lipoprotein and high-density lipoprotein cholesterol levels.^14^ However, a recent retrospective cohort study of over 47 000 adults found that the ketogenic diet has the potential to reduce all-cause mortality, without an adverse effect on cardiovascular-related mortality.^15^ People may experience side-effects while implementing the ketogenic diet, including cravings for carbohydrates, weakness and dizziness. In the Campbell et al study, a total of 12 adverse events (11 minor and 1 serious) were noted in a small sample of 26 recruited participants.^7^ More research on the implementation and long-term safety of the ketogenic diet will be invaluable.

(d) Education. Unlike pharmaceutical treatments supported by large-scale marketing, the ketogenic diet lacks such infrastructure. Educating clinicians on its complexities and monitoring strategies remains challenging. Non-profit organisations, such as the Charlie Foundation, Matthew’s Friends and the International Neurological Ketogenic Society, provide valuable resources, but broader efforts are needed.

(e) Insurance and reimbursement. Insurance rarely covers dietary consultations or coaching, hindering accessibility. At McLean Hospital, attempts to establish an out-patient ketogenic diet consultation service have been stymied by current billing limitations. Advocacy for policy changes and sustainable service models is essential.

(f) Research and education funding. Funding for research on dietary interventions is challenging – philanthropic support has been the primary source of funding to date. The Baszucki Group has invested US$60 million in metabolic psychiatry research; McLean Hospital’s Metabolic and Mental Health Program has received a US$3 million gift from another philanthropist. The field of ketogenic diet research in psychiatry is expanding rapidly, with approximately 20 clinical trials – eight of which are randomised controlled trials – currently under way. However, philanthropic funding alone is unlikely to be sufficient to change entrenched paradigms. Expanding funding sources will be critical to advancing more research, education and implementation strategies.

(g) Complex mechanisms. Evidence-based medicine is often focused on mechanisms of action to support clinical trials. Most psychopharmacologic treatments have clearly established targets and mechanisms of action. The ketogenic diet is more challenging, because it has multifaceted mechanisms that require more comprehensive and systematic investigation. Future studies should focus on optimising protocols, determining therapeutic ketosis levels and systematically measuring metabolic biomarkers to refine its clinical use, establish its efficacy and further understand its complex mechanisms of action.

## A message of hope

The work of Campbell et al and other researchers represents a potential paradigm shift in mental health care. By addressing root metabolic causes, the ketogenic diet offers hope to individuals who have exhausted conventional treatments. This approach is more than a dietary intervention – it is a reimagining of mental health treatment that embraces the intricate interplay between metabolism and brain function.

As research advances, the potential for improving lives through metabolic psychiatry is profound. We owe it to our patients to further investigate these innovative strategies, ensuring that no avenue of hope remains unexplored.
